# Evaluation of the efficacy of mitochondrial fission inhibitor (Mdivi-1) using non-alcoholic steatohepatitis (NASH) liver organoids

**DOI:** 10.3389/fphar.2023.1243258

**Published:** 2023-10-12

**Authors:** Mohamed Elbadawy, Kiwamu Tanabe, Haru Yamamoto, Yusuke Ishihara, Maria Mochizuki, Amira Abugomaa, Hideyuki Yamawaki, Masahiro Kaneda, Tatsuya Usui, Kazuaki Sasaki

**Affiliations:** ^1^ Laboratory of Veterinary Pharmacology, Department of Veterinary Medicine, Faculty of Agriculture, Tokyo University of Agriculture and Technology, Tokyo, Japan; ^2^ Department of Pharmacology, Faculty of Veterinary Medicine, Benha University, Benha, Egypt; ^3^ Department of Pathology, College of Veterinary Medicine, University of Georgia, Athens, GA, United States; ^4^ Faculty of Veterinary Medicine, Mansoura University, Mansoura, Egypt; ^5^ Laboratory of Veterinary Pharmacology, School of Veterinary Medicine, Kitasato University, Aomori, Japan; ^6^ Laboratory of Veterinary Anatomy, Department of Veterinary Medicine, Faculty of Agriculture, Tokyo University of Agriculture and Technology, Fuchu, Japan

**Keywords:** organoids, fibrosis, NASH, mitochondria, ROS, DRP1, liver

## Abstract

Non-alcoholic steatohepatitis (NASH) is known to progress to cirrhosis and hepatocellular carcinoma in some patients. Although NASH is associated with abnormal mitochondrial function related to lipid metabolism, mechanisms for the development and effective treatments are still unclear. Therefore, new approaches to elucidate the pathophysiology are needed. In the previous study, we generated liver organoids from different stages of NASH model mice that could recapitulate the part of NASH pathology. In the present study, we investigated the relationship between mitochondrial function and NASH disease by comparing NASH liver organoids (NLO) and control liver organoids (CLO). Compared with CLO, mitochondrial and organoid morphology was abnormal in NLO, with increased expression of mitochondrial mitogen protein, DRP1, and mitochondria-derived reactive oxygen species (ROS) production. Treatment of NLO with a DPR1 inhibitor, Mdivi-1 resulted in the improvement of morphology and the decreased expression of fibrosis-related markers, *Col1a1* and *Acta2*. In addition, treatment of NASH model mice with Mdivi-1 showed a decrease in fatty liver. Mdivi-1 treatment also prevented fibrosis and ROS production in the liver. These results indicate that NLO undergoes enhanced metabolism and abnormal mitochondrial morphology compared with CLO. It was also suggested that Mdivi-1 may be useful as a therapeutic agent to ameliorate NASH pathology.

## 1 Introduction

With the prevalence of lifestyle-related diseases such as obesity and type 2 diabetes, non-alcoholic fatty liver disease (NAFLD) is now the most frequent chronic liver disease in developed countries. A quarter of the adult population worldwide is affected by NAFLD (Younossi et al., 2016), and its histopathological structures range from lipidosis (benign lipid accumulation) to non-alcoholic steatohepatitis (NASH). NASH is known to cause fatty infiltration of the liver, inflammation of the liver lobules, ballooning, and apoptosis of hepatocytes (Chalasani et al., 2012; Elbadawy et al., 2020a). In NASH, fat toxicity, oxidative stress, and inflammation lead to liver fibrosis, which in some patients progresses to cirrhosis and hepatocellular carcinoma. However, the exact etiology of NASH and therapeutic target molecules for liver fibrosis remains unknown. Therefore, there is a need to develop more precise experimental models that accurately reproduce the pathogenesis of NASH to develop effective therapeutic strategies.

Organoids are 3D cultured tissues made from epithelial cells isolated from organs and can reproduce the cellular composition, structural characteristics, and function of the original tissues at a high level (Sato et al., 2009; Elbadawy et al., 2021a; Elbadawy et al., 2021b; Elbadawy et al., 2021c; Abugomaa et al., 2022; Elbadawy et al., 2022; Yoshida et al., 2022). Compared with traditional 2D cultured cells, the properties of organoids are closer to those of living tissues and organs. Organoids are applied for pathological analysis, drug sensitivity testing, and regenerative medicine (Bartfeld and Clevers, 2017; Elbadawy et al., 2018; Usui et al., 2018; Elbadawy et al., 2019; Abugomaa and Elbadawy, 2020a; Abugomaa et al., 2020; Elbadawy et al., 2020b). In our laboratory, we have successfully established NASH liver organoids from different stages of the NASH-affected mouse model induced by a methionine-choline-deficient (MCD) diet (Elbadawy et al., 2020a). These NASH liver organoids showed the appearance of cells with dendritic-like morphology and the increased expression of liver fibrosis-related genes such as *Collagen type I alpha 1 (Col1a1)*, *Actin alpha 2* (*Acta2*), and others (Elbadawy et al., 2020a). Therefore, the NASH liver organoids can be used as a platform to identify genes that may serve as new diagnostic markers for NASH and to search for new therapeutic agents effective for NASH.

Recently, the multiple parallel hits hypothesis, in which many factors are involved in the pathogenesis of NASH at the same time has been reported (Tilg and Moschen, 2010). The increase of oxidative stress in hepatocytes via the overproduction of reactive oxygen species (ROS) due to the β-oxidation of abundant fatty acids is important in NASH pathogenesis. Also, it has been suggested that mitochondrial dysfunction, which is closely related to lipotoxicity, may be involved in the development of NASH and its progression to cirrhosis and hepatocellular carcinoma (Begriche et al., 2006; Leveille et al., 2019). The damaged hepatocyte mitochondria also induced the activation of hepatic astrocyte and stellate cells and promoted liver fibrosis (An et al., 2020). Mitochondria maintain their dynamics by constantly changing their morphology through a complex of actions such as fission, fusion, and motility to remove damaged sites or complement defective sites (Youle and van der Bliek, 2012). That was mainly orchestrated by optical atrophy 1 (OPA1) which mediates fusion, and dynamin-related protein 1 (DRP1) as well as mitochondrial fission factor (MFF) which regulates fission (Longo et al., 2021).

Aberrancies in these proteins are drivers of NASH development and progression (Zhan et al., 2016). For example, excessive DRP1-triggered mitochondrial fission takes part in apoptosis in various pathological conditions, and thus it has emerged as a promising therapeutic target. A recent study revealed that DRP1-mediated mitochondrial fission promotes carbon tetrachloride-induced liver fibrosis and may function as a therapeutic target for retarding the progression of chronic liver disease (Shan et al., 2022). Mitochondrial division inhibitor 1 (Mdivi-1) has been revealed to play a valuable role in various diseases by inhibiting DRP1-mediated mitochondrial fission (Deng et al., 2021; Ding et al., 2022). MYLS22, a first-in-class and selective inhibitor of OPA1 was shown recently to curtail breast cancer growth by inhibiting OPA1 (Zamberlan et al., 2022). Also, MYLS22 effectively inhibited DRP1 and OPA1 to suppress mitochondrial fission and lowered cardiotoxicity triggered by oxidative stress, hypoxia, hyperglycemia, and poisoning (Zhou et al., 2019). Therefore, we hypothesized that using our established normal liver and NASH organoids (Elbadawy et al., 2020a) to elucidate the relationship between factors related to mitochondrial dynamics in normal liver organoids and NASH organoids could lead to the identification of target molecules for NASH treatment and the development of NASH therapeutic agents.

Based on the above data, we investigated mitochondrial dynamics in normal and NASH liver organoids to clarify the role of mitochondria-related genes in NASH pathogenesis. We also tested whether Mdivi-1 would be effective as a NASH therapeutic agent *in vitro* and *in vivo*.

## 2 Materials and methods

### 2.1 Organoids, chemical staffs, and reagents

Normal liver organoids (CLO) and NASH liver organoids (NLO) established in our previous study (Elbadawy et al., 2020a), were grown up from frozen stocked cryovials and reused for *in vitro* experiments. The culture medium and conditions were the same as previously described (Broutier et al., 2016; Elbadawy et al., 2020a). DRP1 inhibitor (Mdivi-1, M3108; Tokyo Kasei Kogyo Co., Ltd., Tokyo) and OPA1 inhibitor (MYLS22, S9885; Selleck chem.com, USA) were obtained commercially. To check ROS production by organoids, a solution of mitochondrial superoxide (mtSOX) Deep Red (Dojindo Laboratories, Kumamoto, Japan) was used. Primary antibodies were as follows: DRP1, MFF, and OPA1 were from Cell Signaling Technology, Inc., (Danvers, Massachusetts, United states), α-SMA was from DAKO (Glostrup, Denmark), β-actin was from MilliporeSigma (Burlington, Massachusetts, United states). Secondary antibodies were HRP-conjugated anti-rabbit IgG (Cayman, Ann Arbor, Michigan, United states) and HRP-conjugated anti-mouse IgG (MilliporeSigma). MCD diet and standard pelleted control diet were purchased from Oriental East Co., Ltd. (Tokyo, Japan).

### 2.2 Animals

Twenty-four male C57BL/6 mice of 7-weeks-old were purchased (Oriental East Co., Ltd.) and used for *in vivo* experiments. During acclimatization, the mice were housed in three mice per cage with a 12-h light-dark cycle in a temperature and ventilation-controlled room with *ad libitum* access to food and water until the experiment begins. NASH mice were fed an MCD diet to induce the NASH state, while the control mice were fed a standard pelleted mice diet. MCD was administered to mice for 12 weeks to induce a severe NASH state model (Itagaki et al., 2013). This experiment was conducted with the approval of the Tokyo University of Agriculture and Technology (TUAT) Animal Care and Use Committee and the Ethics Committee (R04-121).

### 2.3 Experimental design

The study was carried out *in vitro* and *in vivo*. The *in vitro* experiments were carried out on CLO and NLO generated in previous studies (Broutier et al., 2016; Elbadawy et al., 2020a) to compare morphology, microstructure, ROS production level, and protein expression level of mitochondria-related fission and fusion markers (DRP1, MFF, and OPA1). Further, the effects of Mdivi-1 on NLO size, morphology (dendritic shape formations), and expression level of NASH markers including *Col1a1*, *Acta2*, and *DRP1* were investigated. The *in vivo* experiments were conducted on mice to elucidate the ameliorative effects of Mdivi-1 on NASH state using different analyses.

#### 2.3.1 Organoid culture

CLO and NLO were grown up in Matrigel (BD Bioscience, San Jose, CA, United states) and stem cell stimulating media. After the cryovials were thawed at 37°C, the organoids were washed once with phosphate buffer saline (PBS) and centrifuged at 200 ց for 3 min. The organoid pellets were gently suspended in Matrigel on ice and dropped in 24-well culture plates (40 µL/well). The plates were then incubated in a CO_2_ incubator for 30 min to solidify the gel, and a stem cell stimulating medium was used to grow organoids. The culture media and conditions were used as before (Broutier et al., 2016; Elbadawy et al., 2020a), and the organoids were passaged every 7–14 days using 5 mM EDTA/PBS and TrypLE™ Express enzyme (1X solutions, Gibco, Life Technologies Co., Grand Island, NY, United States) at 1:2–4 split. The organoids were used to compare morphology (bright field and ultrastructure), ROS production, and protein expression level of DRP1, MFF, and OPA1. The liver organoids generated from mice fed with normal or MCD diet and administered Mdivi-1 or vehicle were generated, and cultured as described before (Elbadawy et al., 2020a) to analyze the modulating effects of Mdivi-1 on the size of NLO using ImageJ software (National Institutes of Health), and the mean value was calculated.

#### 2.3.2 Morphology of CLO and NLO

After the appropriate growth of organoids, phase contrast images of CLO and NLO were captured under an optical microscope (CKX-53; Olympus Corporation, Tokyo, Japan) to check the morphology of organoids.

#### 2.3.3 Treatment of NLO with inhibitors of mitochondrial fission or fusion factor

After 7–14 days of culture, NLO were used to assess the effect of mitochondrial fission or fusion factor. To dissolve Matrigel, five hundred µL of 5 mmol/L EDTA/PBS was added per well and the culture plate was put on ice for 90 min. The organoid suspension was gathered into a 15-mL tube and centrifuged at 600 *g* for 3 min. Organoid pellets were washed with PBS and trypsinized using TrypLE™ Express enzyme (1X solutions) at 37°C for 5 min. Thereafter, a vigorous pipetting was conducted to dissociate organoids into single cells. The solution was passed through a 70 μm cell strainer (Falcon, Cary, NC, United States), and seeded in triplicate in Matrigel at 1×10^5^ cells onto 24-well plates. Twenty-4 h later, the seeded NLO were treated with 50 µM of Mdivi-1 or MYLS22. For control wells, the same volume of DMSO (Fujifilm Wako Pure Chemicals Co., Ltd.) was added to the culture media, and plates were incubated for 72 h in a CO_2_ incubator. Thereafter, solutions were aspirated, and the same treatments were added and incubated again for 72 h. The organoids were then photographed several times with an optical microscope (CKX-53; Olympus Corporation), the number of elongated dendritic-like forms present per field of view was counted, and the average value was quantified. Also, the diameter of spherical organoids was measured using ImageJ software (National Institutes of Health), and the average value was calculated.

#### 2.3.4 Analysis of the effects of Mdivi-1 on free fatty acid (FFA)-induced lipid accumulation in NLO

After 7–10 days of NLO culture, lipid accumulation was induced by mixing the culture medium with 2 mM oleic acid (Sigma-Aldrich) with or without 50 µM Mdivi-1 for 48 h. The oleic acid solution was prepared as described before (McCarron et al., 2021; Thompson and Takebe, 2020). After dissolving gel, organoids were carefully harvested, washed once with cold PBS, and fixed in 4% PFA at RT for 60 min. For lipid staining, organoids were washed twice with PBS and incubated with the culture medium containing LipidTOX (1:200, Thermo Fisher) and DAPI (1:1000, Dojindo) for 30 min at RT. Multiple images were captured from different fields using an all-in-one BZ-X800 Keyence Fluorescence Microscope (Osaka, Japan). The fluorescence intensity of three different fields was quantified using ImageJ software (National Institutes of Health).

### 2.4 Analysis of the *in vivo* effects of Mdivi-1 on NASH condition

This experiment was conducted with the approval of the TUAT Animal Care and Use Committee and the Ethics Committee (R04-121). Twenty-four 7-weeks-old male C57BL/6 mice were divided into four groups (Figure 3A). The first group was assigned as a control group and fed a normal diet and administered vehicle (Mdivi-1 solvent). The second one was assigned as control-treated and fed a normal diet and administered Mdivi-1. The third group was assigned as NASH-non-treated and fed an MCD diet and administered vehicle. The fourth group was assigned as NASH-Mdivi-1-treated and fed an MCD and administered Mdivi-1. Mdivi-1 injection solution was prepared by dissolving the powder in DMSO and diluting it in physiological saline (Otsuka Pharmaceutical Factory, Inc.). Mdivi-1 was administered intraperitoneally to the assigned groups (second and fourth ones) at a dose of 1 mg/kg of body weight every other day for 8 weeks (Ding et al., 2022). MCD diet was administered to the NASH-assigned groups (third and fourth ones) for 8 weeks as indicated by the manufacturer. At the end of the experiments, mice were euthanized under isoflurane anesthesia, weighed, and livers were dissected and washed with PBS and weighed. Blood samples were collected and centrifuged at 2000 *g* for 15 min at 4°C and the serum was collected for liver functions-related biochemical analyses. Samples from liver tissues were picked up to generate organoids and check their sizes. Other samples were used for the analyses of histopathology, microstructure by Transmission Electron Microscopy (TEM), oil red o staining, Masson trichrome staining, the extractions of RNA and protein.

#### 2.4.1 Serum biochemical analyses

Serum samples collected from differentially treated mice were used to measure the serum concentration of liver function-related parameters [Alanine aminotransferase (ALT), Aspartate aminotransferase (AST), and total cholesterol (T-CHO)] and Triglyceride (TG) through ORIENTAL EAST CO., LTD., Tokyo, Japan.

#### 2.4.2 Hematoxylin and eosin (H&E) staining

The excised liver tissue was fixed with 4% paraformaldehyde (PFA) for 24 h and embedded in paraffin. Paraffin blocks containing liver tissue were sliced to 5 µm thickness by a sliding microtome (REM700; Daiwa Koki Kogyo, Saitama, Japan) and paraffin sections were prepared. Sections were deparaffinized and stained with H&E following the standard procedures. Images were captured with an optical microscope (BX-43; Olympus Corporation).

#### 2.4.3 Masson’s trichrome staining

The procedure was performed according to the manufacturer’s protocol (Muto Pure Chemical Co., Ltd., Tokyo, Japan). Sections were deparaffinized and treated with the first mordant solution for 20 min. Next, the nuclei were stained using Weigertʼs iron hematoxylin solution for 10 min. The sections were then washed with a running tap water stream for a few min and treated with the second mordant for 30 s, 0.75% orange G solution for 1 min, and washed with 1% acetic acid solution. The nuclei were then immersed in Masson’s dye B for 20 min, washed with 1% acetic acid solution, and immersed in 2.5% phosphotungstic acid solution for 20 min. After washing with 1% acetic acid solution again, the sections were immersed in aniline blue dye for 10 min to stain collagen fibers. Images were taken with an optical microscope (BX-43; Olympus Corporation). The blue-colored area was quantified using ImageJ software.

### 2.5 Transmission electron microscopy (TEM)

For analysis of the microstructure of CLO and NLO, and liver tissues from differentially-treated mice, TEM was used as described previously (Elbadawy et al., 2021c). Briefly, at an appropriate growth level, the CLO and NLO pellets or liver sections were fixed with 2.5% glutaraldehyde for 3 h at room temperature (RT) in 0.1% cacodylate (pH 7.4). Thereafter, organoids and liver tissues were washed with 0.1 M cacodylate (pH 7.4), incubated in 2% osmium tetroxide and 1.5% K_4_Fe(CN)_6_ in 0.1 M sodium cacodylate (pH 7.4) for 2 h at 4°C, and washed with distilled water. The organoids and tissues were then dehydrated with graded ethanol solutions (50%, 70%, 80%, 90%, 95%, and 99.5 up to 100%) and embedded in Epon. Ultrathin sections of 70–110 nm size were prepared with a diamond knife on a Leica UC7 ultramicrotome and transferred onto 50-mesh copper grids covered with a form bar and carbon film. The sections were post-stained with uranyl acetate for 15 min at RT and lead citrate. Sections were imaged using a transmission electron microscope (H-7500, Hitachi, Tokyo, Japan) using a TEM digital camera (NanoSprint500, Hitachi).

### 2.6 Assay of mitochondria-derived ROS production

To measure ROS production in CLO and NLO, the Matrigel domes containing organoids were dissolved on ice using 5 mM EDTA/PBS solution for 90 min. The organoids solution was collected in a 15 mL tube and centrifuged (600 *g*/3 min/4°C), and the supernatant was aspirated. The organoids were washed once with PBS, trypsinized for 5 min in a water bath (37°C), filtered using a 70-μm cell strainer (Falcon, Cary, NC, United States), and seeded at 5 × 10^4^ cells/well. A special 2.5D culture medium (Elbadawy et al., 2020a) was added and the plates were incubated overnight in a 5% CO_2_ incubator at 37°C. In next day, the medium was aspirated, a solution of mtSOX Deep Red was mixed with the medium to 10 μmol/L, and 1 μg/mL Hoechst (Fujifilm Wako Pure Chemicals Corporation) was added. The plates were then placed in a 5% CO_2_ incubator at 37°C for 30 min. Thereafter, the solution was aspirated, PBS was added, and images were captured. To assess ROS production level in the liver sections of differentially treated mice, sections were washed with PBS, placed in a 10 μmol/L solution of mtSOX Deep Red in PBS with 1 μg/mL Hoechst, and placed in a 37°C, 5% CO_2_ incubator for 30 min. Images of organoids and liver sections were then captured using a fluorescence microscope (BX52; Olympus Corporation) and the DP2-BSW program (Olympus Corporation). The fluorescence intensity of three different fields was quantified using ImageJ software (National Institutes of Health).

### 2.7 Western blotting

The protein expression was examined using Western blotting as described previously (Usui et al., 2018; Elbadawy et al., 2021b). Briefly, the Matrigel dome-containing organoids were melted on ice for 90 min using 5 mM EDTA/PBS. The organoid solutions were collected and centrifuged, and the supernatants were aspirated. Cell lysis buffer (MilliporeSigma) with 1% protease inhibitor (Sigma-Aldrich) was added to the organoids pellets and the minced fragments from precooled liver tissues in liquid nitrogen, which were pipetted and set on ice for 15 min. The protein lysates were then centrifuged at 12,000 *g* for 10 min, and the supernatant was collected, labeled, and kept at −80°C until analysis. Protein concentrations in lysates were measured using the DC protein assay kit (Bio-Rad Laboratories, Hercules, California, United States) at a wavelength of 650 nm and quantified.

Electrophoresis was then performed on a 10% polyacrylamide gel (Fujifilm Wako Pure Chemicals Co., Ltd.). The protein solution was mixed with 4X SDS sample buffer (Bio-Rad Laboratories) at 3:1 (v/v) and heated at 95°C for 5 min. Equal amounts of protein (10 µg) were loaded into the gel, electrophoresed at 150 V, 400 mA for 60 min, and transferred to a PVDF membrane (WSE-4051; ATTO Corporation, Tokyo, Japan) for blotting. After blocking, the membranes loaded by protein lysates from non-treated CLO and NLO were incubated with primary antibodies (DRP1; 1:500, MFF; 1:500, and OPA1; 1:500). The membranes loaded by protein lysates from Mdivi-1-treated NLO were incubated with antibody to α-SMA (1:500). The membranes loaded protein lysates from liver tissues were incubated with antibody to α-SMA (1:500). Incubation was performed overnight at 4°C. The membranes were then washed three times with 0.1% TBS-T for 5 min and treated with HRP-conjugated anti-rabbit IgG (1:5000) or HRP-conjugated anti-mouse IgG (1:5000) for 1 hour at RT, followed by washing three times with 0.1% TBS-T again for 5 min. After treatment with Immobilon Forte Western HRP Substrate (MilliporeSigma), chemiluminescence of bands was observed using LAS3000 (Fujifilm Corporation, Tokyo, Japan) and photographed. The images were quantified using ImageJ software (National Institutes of Health).

### 2.8 Quantitative real-time PCR

Quantitative real-time PCR was performed as described previously (Elbadawy et al., 2020a). Briefly, RNA samples were prepared from Mdivi-1- or DMSO-treated NLO and minced fragments from precooled liver tissue in liquid nitrogen using the NucleoSpin RNA kit (MACHEREY-NAGEL, Düren, Germany). The RNA was then converted to cDNA using the ReverTra Ace qPCR RT Kit (Toyobo Co., Ltd., Osaka, Japan). The PCR was then performed on cDNA using the QuantiTect SYBR I kit (Qiagen, Hilden, Netherlands) and the StepOnePlus Real-Time PCR system (Applied Biosystems, Waltham, MA, United States). Using the 2^−ΔΔCT^ method, values of cycle threshold (Ct) obtained in quantification were used for calculations of fold changes in mRNA abundance. The specific primers (Fasmac Corporation, Kanagawa, Japan) used for experiments were shown in Table 1.

**TABLE 1:** Primers for real-time quantitative PCR analysis.

	Primer	Sequence
*Collagen I*	Forward	5′-AAG​GCA​ATG​CTG​AAA​TGT​CC-3′
	Reverse	5′-ATG​TCC​CAG​CAG​GAT​TTG​AG-3′
*Ldlr*	Forward	5′-GGG​CCT​CTG​TCT​GGT​GTT​TA-3′
	Reverse	5′-AGC​AGG​CTG​GAT​GTC​TCT​GT-3′
*Lpl*	Forward	5′-GGG​CTC​TGC​CTG​AGT​TGT​AG-3′
	Reverse	5′-CCA​TCC​TCA​GTC​CCA​GAA​AA-3′
*Pnpla3*	Forward	5′-GAA​CCA​CTG​CAA​GGT​TTG​GT-3′
	Reverse	5′-CCT​TCA​GTG​CTG​AGG​TGT​CA-3′
*Tm6sf2*	Forward	5′-ACG​GAC​ATT​CGG​AGA​AAC​TG-3′
	Reverse	5′-GGG​CAT​TAG​AGT​CTG​GGT​GA-3′
*α-sma*	Forward	5′-AGG​GCT​GGA​GAA​TTG​GAT​CT-3′
	Reverse	5′-GCC​AGC​AAA​GGT​CAG​AGA​AG-3′

### 2.9 Statistical analysis

Data are presented as mean ± SEM. Statistical evaluation was performed by one-way analysis of variance (ANOVA) and followed by the Bonferroni’s test. *p* values were considered significantly different at *p <* 0.05.

## 3 Results

### 3.1 Structures and functions of mitochondria in NASH liver organoids

**FIGURE 1:**
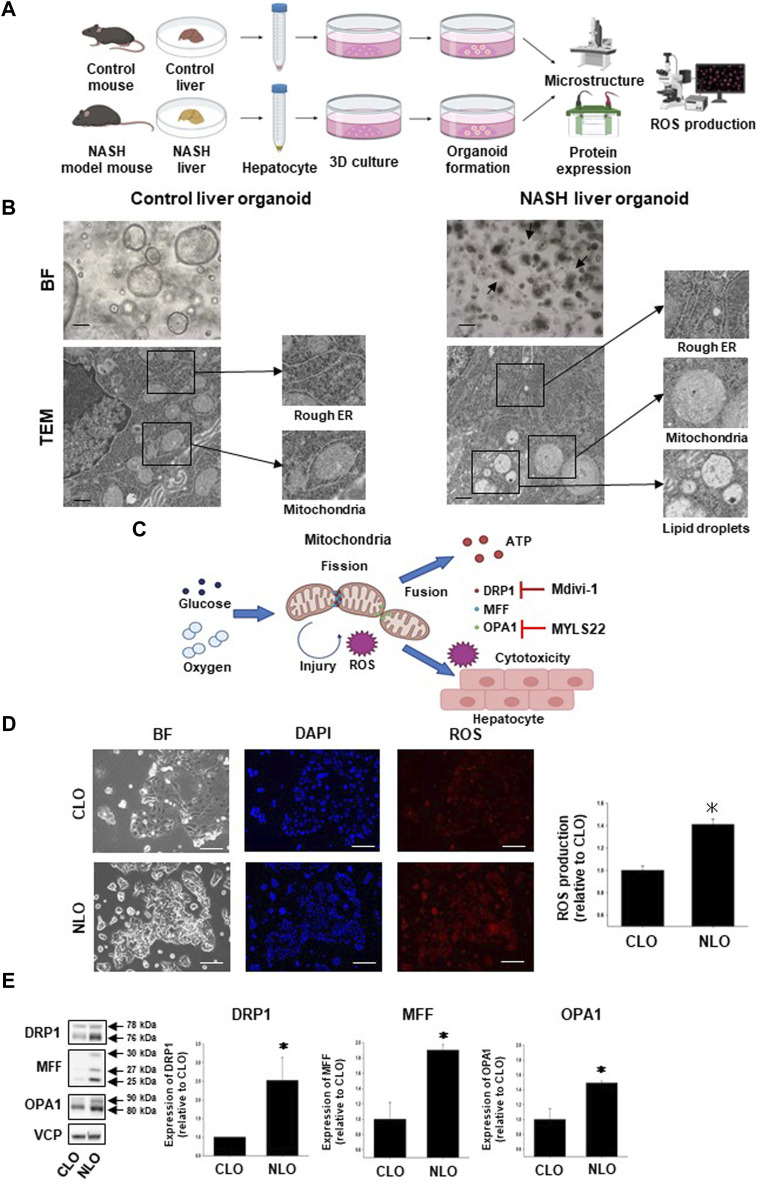
Structures and functions of mitochondria in non-alcoholic steatohepatitis (NASH) liver organoids. To generate and analyze NASH liver organoids (NLO), liver tissues were harvested from NASH model mice induced by feeding a high-fat diet, not including methionine and choline for 12 weeks **(A)**. Bright-field (BF) and transmission electron microscopy (TEM) images of control and NASH liver organoids. BF: Scale bar: 100 μm, TEM: Scale bar: 600 nm **(B)**. Arrows show the typical dendritic-like cells **(B)**. Schematic diagram of the relationship between mitochondrial fission/fusion and reactive oxygen species (ROS) in NASH liver tissues. Glucose and oxygen stimulate mitochondria-derived ROS production, which induces cytotoxicity of the hepatocyte. Mdivi-1 inhibits mitochondrial division factor (DRP1), while MYLS22 inhibits mitochondrial fusion factor (OPA1) **(C)**. Mitochondria-derived ROS production in NLO. Representative images for mtSOX Deep Red staining of control and NASH liver organoids (CLO and NLO). Scale bar: 100 μm **(D)**, (n = 3). Fluorescence intensity in the stained images was quantified by using ImageJ software. Results were shown as fold increase relative to CLO and expressed as mean ± S.E.M. *p < 0.05 vs. CLO **(E)**. Protein expression level of DRP1, MFF, and OPA1 was compared between CLO and NLO as determined by Western blotting. Equal loading of protein was confirmed by using a total Valosin-containing protein (VCP) antibody. Quantification of protein expression level was analyzed by ImageJ software (E, n = 3–5). Results are expressed as mean ± S.E.M. *p < 0.05 vs. CLO.

In the previous study, we generated CLO and NLO from C57BL/6 mice, which were fed a normal or MCD diet for 12 weeks (Elbadawy et al., 2020a). In the present study, we first compared the structures of the intracellular organelle between CLO and NLO using TEM (Figure 1A). As reported previously, CLO showed large spherical structures, while NLO showed small spherical structures with epithelial-mesenchymal transition and elongated dendritic- or stellate-like cell morphology (Figure 1B). The microstructures of NLO showed an increase in the rough endoplasmic reticulum (ER), fat droplets, and abnormal mitochondrial morphology (become swollen and spherical) compared with CLO (Figure 1B, Supplementary Figure S1).

To investigate the detailed relationship between the disease progression of NASH and mitochondria-related signals, we next compared the mitochondria-derived ROS production and expression level of mitochondria fission/fusion proteins (DRP1, MFF, and OPA1) between CLO and NLO (Figures 1A, C). We observed that ROS production was significantly increased in NLO compared with CLO (Figure 1D). Protein expression levels of DRP1, MFF, and OPA1 were also significantly increased in NLO compared with CLO (Figure 1E). These results suggest that abnormal functions and structures of mitochondria might increase ROS production in NLO.

### 3.2 Effects of inhibitor of mitochondria fusion or fission protein on fibrosis of NASH liver organoids

**FIGURE 2:**
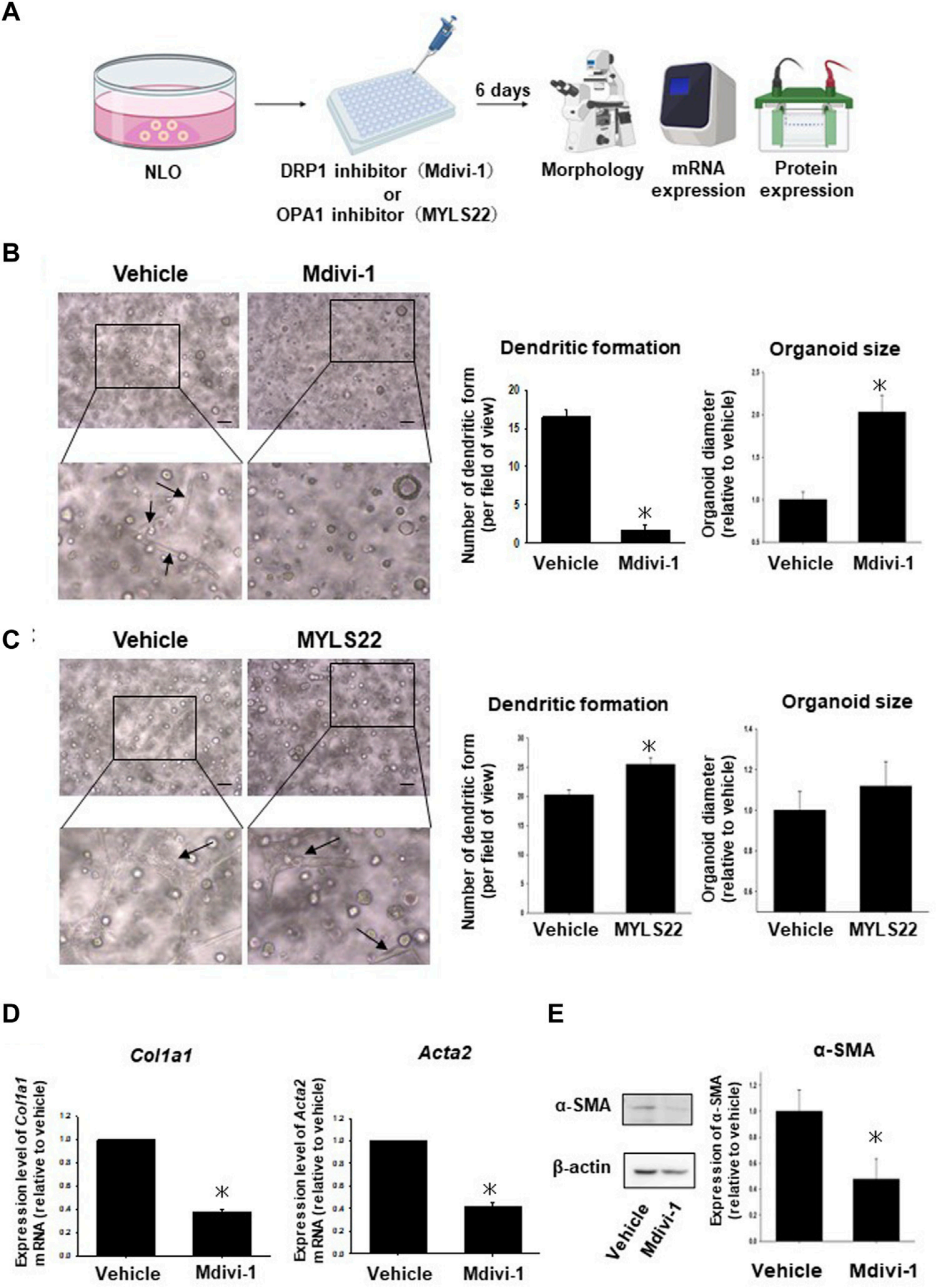
Effects of inhibitor of mitochondria fusion or fission protein on NLO. After NLO were treated with Mdivi-1 (50 µM) or MYLS22 (50 µM) for 6 days, organoid morphology, mRNA, and protein expression levels were analyzed **(A)**. Phase contrast microscopic images of NLO treated with Mdivi-1 **(B)** or MYLS22 **(C)**. Dendritic-like cells and organoid size were quantified by using ImageJ software (n = 4). Arrows show the typical dendritic-like cells **(B,C)**. Expression of fibrosis-related genes, *Col1a1,* and *Acta2* mRNA in NLO treated with Mdivi-1 was determined by quantitative real-time PCR **(D)**. The expression level of each gene was quantified based on the ratio of expression level to *GAPDH* and shown as a fold increase relative to vehicle (n = 4). Results were expressed as mean ± S.E.M. *p < 0.05 vs. vehicle **(D)**. Protein expression level of α-SMA in NLO treated with Mdivi-1. The expression level of α-SMA was analyzed as determined by Western blotting **(E)**. Equal loading of protein was confirmed by total actin antibody. Quantification of protein expression level was analyzed by ImageJ software (n = 3). Results are expressed as mean ± S.E.M. *p < 0.05 vs. Vehicle.

To investigate the relationship between mitochondria-related molecules and fibrosis-related organoid morphology, NLO were treated with a mitochondrial mitogen (DRP1) inhibitor, Mdivi-1, or a fusion factor (OPA1) inhibitor, MYLS22 for 6 days (Figure 2A). In NLO, dendric-like cells and each organoid gradually increased after seeding the organoid cells. Mdivi-1 treatment significantly decreased the number of dendritic-like cells and increased the size of spherical structures (Figure 2B). On the other hand, MYLS22 treatment significantly increased the number of dendritic-like cells and had no effects on the size of spherical structures (Figure 2C). These results imply that DRP1 but not OPA1 has an important role for morphological changes of NLO.

### 3.3 Effects of Mdivi-1 treatment on expression of fibrosis-related genes in NASH liver organoids

In the previous study (Elbadawy et al., 2020a), the protein expression analysis of fibrosis-related proteins, Col1a1 and Acta2, in NLO was confirmed. To confirm the effects of Mdivi-1 treatment on the expression of fibrosis-related genes in NLO, mRNA expression levels of fibrosis-related markers (*Col1a1 and Acta2*) were analyzed by real-time quantitative PCR. As expected, Mdivi-1 treatment significantly reduced expression levels of *Col1a1 and Acta2* compared with vehicle treatment (Figure 2D). In NLO, the protein expression level of Acta2 protein (α-SMA) was also inhibited by Mdivi-1 treatment compared with vehicle treatment (Figure 2E).

### 3.4 Effects of Mdivi-1 on FFA-induced lipid accumulation in NLO

High level of serum FFA is the main contributor to fatty liver disease and is elevated in NAFLD and NASH patients (Zhang et al., 2014). We thus checked the effect of Mdivi-1 on lipid accumulation in NLO after loading it with oleic acid. After treating NLO with 2 mM oleic acid, they showed a progressive lipid accumulation as visualized by LipidTOX staining compared with vehicle treatment (Supplementary Figures S2A, B). Treatment with Mdivi-1 significantly decreased the oleic acid-induced lipid accumulation in NLO (Supplementary Figures S2A, B).

### 3.5 Effects of long-term Mdivi-1 administration to mice on the development of NASH disease

**FIGURE 3:**
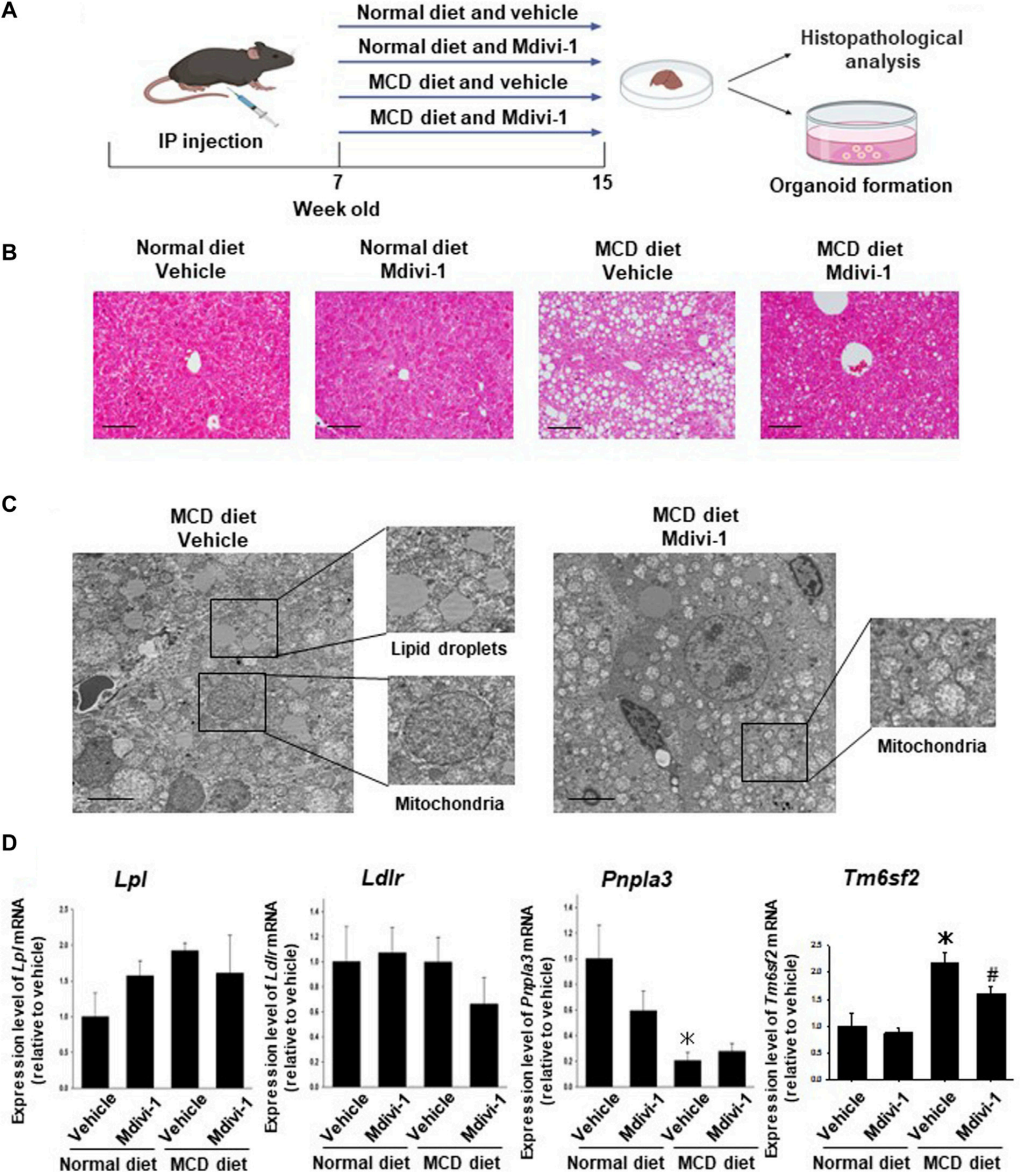
Effects of long-term Mdivi-1 administration to mice on the development of NASH disease. Mdivi-1 was intraperitoneally administered for 8 weeks while feeding a normal diet or a methionine-choline deficient (MCD) diet. Liver tissues were then isolated for histopathological analysis and preparation of organoid culture **(A)**. H&E staining images of the liver tissues from each group of mice **(B)**. Scale bar: 100 µm. Effects of long-term Mdivi-1 administration to mice on the microstructure of liver tissues **(C)**. Liver tissues from each group of mice were observed by using TEM. Boxes show typical lipid droplets and mitochondria in the liver tissues. Scale bar: 10 μm. Expression of lipid metabolism-related genes, *Lpl, Ldlr, Pnpla2,* and *Tm6sf2* mRNA in liver tissues from each group of mice was determined by quantitative real-time PCR **(D)**. The expression level of each gene was quantified based on the ratio of expression level to *GAPDH* and shown as a fold increase relative to the control mice (n = 4–5). Results were expressed as mean ± S.E.M. *p < 0.05 vs. vehicle with normal diet. #*p* < 0.05 vs. vehicle with MCD diet.

To check the effects of Mdivi-1 *in vivo*, seven-weeks-old C57BL/6 mice were fed MCD diet for 8 weeks and Mdivi-1 was administered (Figure 3A). In MCD diet-fed mice, body and liver weight was significantly lower than control mice, which was not affected by Mdivi-1 administration (Supplementary Figure S3A). The serum concentration of ALT but not AST in NASH mice was inclined to be higher than control mice (Supplementary Figure S3B), while total cholesterol (T-CHO) and triglycerides (TG) were significantly lower (Supplementary Figure S3B). Mdivi-1 administration had no effects on these parameters. On the other hand, histological analysis showed that the accumulation of lipid droplets of the liver tissues from Mdivi-1-administered and MCD diet-fed mice was decreased compared with vehicle-administered and MCD diet-fed mice (Figure 3B). Observation by TEM also showed Mdivi-1 administration decreased accumulation of lipid droplets and abnormal mitochondrial morphology in the liver tissues from MCD diet-fed mice (Figure 3C). To investigate the inhibitory mechanisms of Mdivi-1, we next checked expression levels of lipid metabolism-related genes (*Lpl, Ldlr, Pnla3,* and *Tm6sf2*). Among these genes, Mdivi-1 significantly decreased *Tm6sf2* expression, which was significantly upregulated in the liver tissues of MCD diet-fed mice (Figure 3D).

### 3.6 Effects of long-term Mdivi-1 administration to mice on liver fibrosis

**FIGURE 4:**
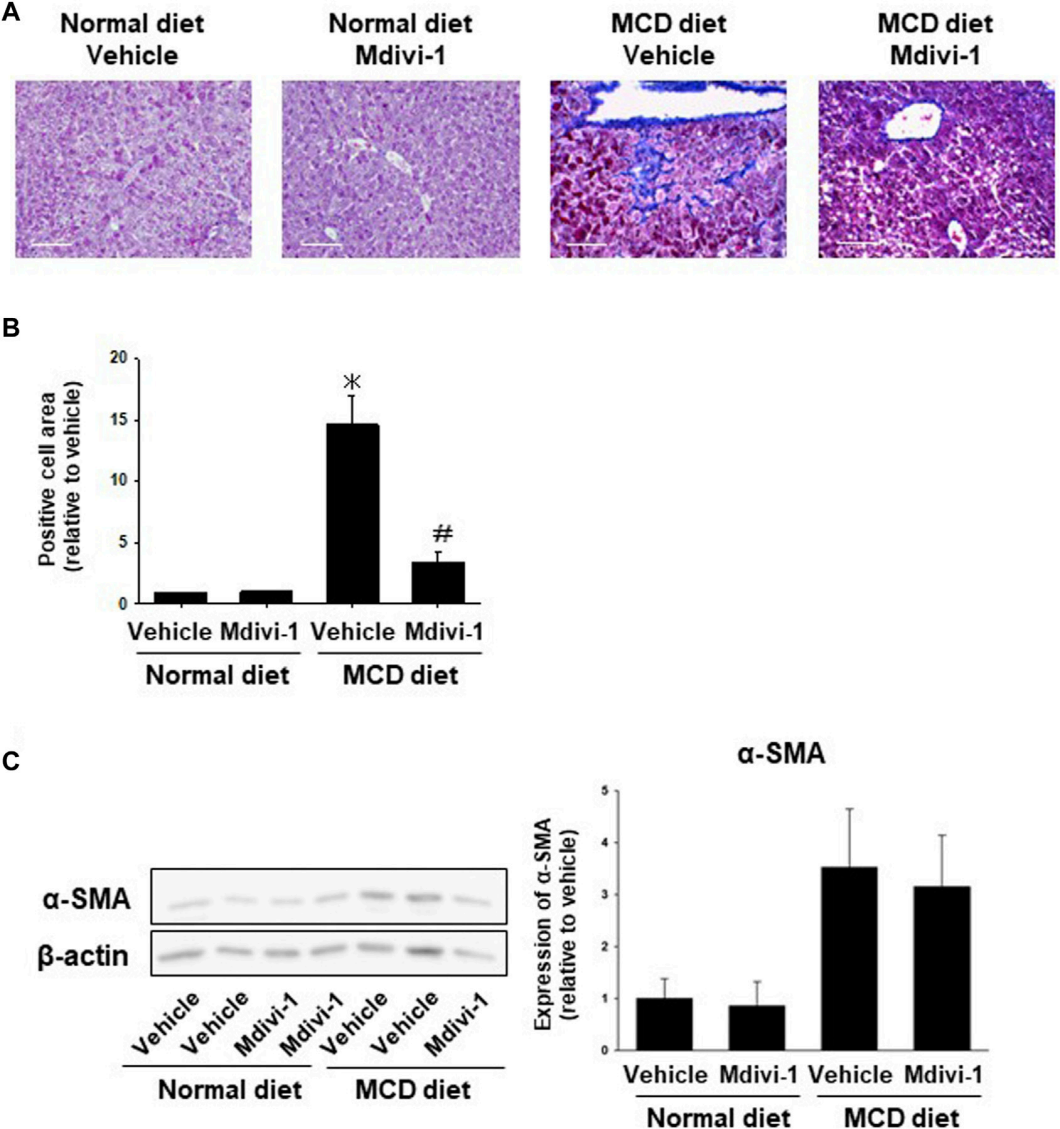
Effects of long-term Mdivi-1 administration to mice on liver fibrosis. Representative images for Masson’s Trichrome staining of liver tissues from each group **(A)**. Scale bar: 100 μm. In the Masson’s Trichrome staining, the blue-colored area representing fibrosis and collagen deposition was quantified and shown as a fold increase relative to control **(B)**, (n = 3). Expression level of α-SMA protein was analyzed by Western blotting **(C)**. Equal loading of protein was confirmed by total actin antibody. Quantification of protein expression level was analyzed by ImageJ software (n = 5–6). Results are expressed as mean ± S.E.M. *p < 0.05 vs. vehicle with normal diet.

We next checked whether Mdivi-1 prevents fibrosis in NASH mice. Masson trichrome staining showed that collagen fibers were observed in the peri-central vein and sinusoids in the liver tissues from MCD diet-fed mice, which was significantly inhibited by Mdivi-1 administration (Figures 4A, B). To confirm the inhibitory mechanisms by Mdivi-1, we checked expression levels of α-SMA. Western blotting analysis also showed that the expression level of α-SMA was higher in the liver tissues from MCD diet-fed mice, which was slightly prevented by Mdivi-1 administration (Figure 4C). Mdivi-1 also decreased *Acta2* but not *Col1a1* expression, which was slightly upregulated in the liver tissues of MCD diet-fed mice (Supplementary Figure S4).

### 3.7 Effects of long-term Mdivi-1 administration to mice on mitochondria-derived ROS production

**FIGURE 5:**
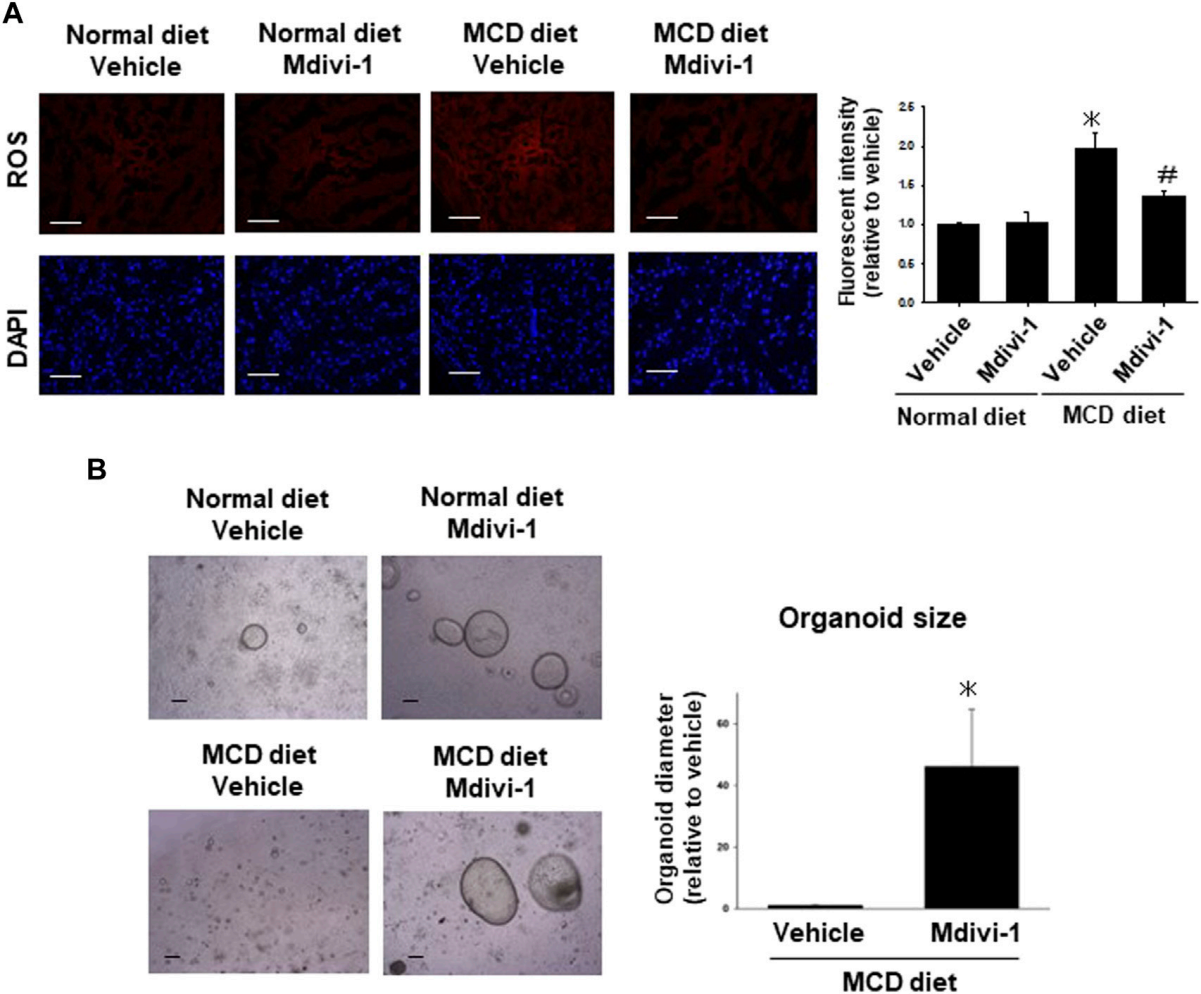
Effects of long-term Mdivi-1 administration to mice on ROS production and capacity of organoid formation. Representative images for mtSOX Deep Red staining of liver tissues from each group of mice **(A)**, (n = 4). Scale bar: 100 μm. Fluorescence intensity in the stained images was quantified by using ImageJ software. Results were shown as fold increase relative to the control mouse and expressed as mean ± S.E.M. *p < 0.05 vs. vehicle with a normal diet. The representative images of organoids were taken after seeding the same number of cells isolated from the liver tissues of each group of mice **(B)**. Organoid sizes (n = 4) of MCD diet group at day 5 were quantified by using ImageJ software. Results were shown as a fold increase relative to MCD diet-fed mice and expressed as mean ± S.E.M. *p < 0.05 vs. vehicle with MCD diet.

To analyze whether the long-term Mdivi-1 administration to mice affects mitochondria-derived ROS production in liver tissues from MCD diet-fed mice, the liver tissues from each group of mice were stained for mtSOX Deep Red. ROS production was significantly higher in the liver tissues from MCD diet-fed mice, which was significantly inhibited by Mdivi-1 administration (Figure 5A).

### 3.8 Effects of long-term Mdivi-1 administration to mice on the capacity of organoid formation

To finally check the effects of Mdivi-1 on the capacity of organoid formation, the efficacy of organoids was evaluated by organoid size at day 5 after seeding the same number of cells (Figure 5B). Organoid size was smaller in the liver tissues from MCD diet-fed mice compared with control mice, which was significantly improved by Mdivi-1 administration (Figure 5B). Considering these results, Mdivi-1 administration might prevent the development of NASH diseases through maintenance of functional hepatocytes in NASH mouse.

## 4 Discussion

NASH model can be induced by feeding mice fast food (FF) diet (Charlton et al., 2011), high-carbohydrate diet (HCD) (Prisingkorn et al., 2017), high-fat diet (HFD) (Zarzour et al., 2018), or MCD diet (Lan et al., 2021). Further, some models showed that the advanced stage of NASH (with fibrosis) was reached by a second stimulus such as tunicamycin (Kim et al., 2018), dexamethasone (Rahimi et al., 2020), or carbon tetrachloride (Tsuchida et al., 2018; Zhang et al., 2020). Unlike the HFD, the MCD-fed mice model presents the histological hallmark of NASH with a transition from simple steatosis to steatohepatitis to fibrosis in a short time (Peng et al., 2020; Im et al., 2021) with unrecovered histological alterations as compared with HFD-fed ones after withdrawal from the tested diet (Itagaki et al., 2013). However, the MCD shows lower insulin resistance levels (Rinella and Green, 2004) compared with HFD (Lee et al., 2016). Although a lot of studies about the cellular and molecular pathogenicity of NASH have been carried out using these dietary animal models, further studies on gene expression could present additional approaches to elucidate the sophisticated pathogenesis of NASH.

Mitochondrial dysfunction in the liver was reported in NASH pathophysiology (Xu et al., 2021). In addition to fat accumulation, alterations in mitochondrial morphology and dynamics were observed in patients with metabolic syndrome that participate in NASH development (Egan et al., 2011; Einer et al., 2018). At the organoid level, McCarron et al. showed that lipid accumulation in the patient-derived NASH organoids was about 4.5-fold higher compared with the average of healthy organoids (McCarron et al., 2021). In another study, exposure of human-induced pluripotent stem cell (iPSC)-derived liver organoids-on-a-chip system to free fatty acids revealed that organoids showed accumulation of lipid droplets and triglycerides with the upregulated expressions of lipid metabolism-related genes, indicating the abnormal lipid metabolic process (Wang Y. et al., 2020; Hendriks et al., 2023). Mitochondria are structurally characterized by a spherical or elongated ovoid shape (Palade, 1952). Ultrastructural alterations such as giant mitochondria, loss of cristae, and the presence of linear crystalline inclusions within the mitochondrial matrix of an increased electron density were reported in NASH patients (Lotowska et al., 2014; Wang Z. et al., 2020; Shami et al., 2021). In the present study, NLO microstructure showed fat accumulation and mitochondrial swelling and deformation (Figure 1B). This data suggests that NLO mimics the microstructure of liver tissue in NASH pathology.

Oxidative stress is caused by a conflict between ROS production and antioxidant defense resulting in DNA and tissue damage (Sies, 2015; Abugomaa and Elbadawy, 2020b). In NASH pathology, excessive fat accumulation in hepatocytes impairs mitochondrial oxidative capacity (Delli Bovi et al., 2021), and increased ROS production (Rolo et al., 2012) which in turn contributes to the activation of stellate cells and with upregulation of proinflammatory cytokines (TNF-α, IL-1, and IL-6), apoptosis, and development of fibrosis (Delli Bovi et al., 2021; Zhou et al., 2022). Exposed iPSC-derived liver organoids-on-a-Chip system to free fatty acids increased ROS production and upregulated inflammatory cytokine-related genes, and fibrogenic markers (Wang et al., 2020). In the present study, ROS production was significantly increased in NLO (Figure 1D) and liver tissues from NASH mice (Figure 5A), verifying their role in the pathogenesis of NASH.

The mitochondria maintain their functions by removing damaged sites and complementing defective sites through frequent fission and fusion that are regulated by DRP1, MFF, and OPA1 proteins (Youle and van der Bliek, 2012). Alterations in these proteins drive NASH development and progression (Zhan et al., 2016). In Western diet-induced NASH mice models, DRP1 expression was reduced (Krishnasamy et al., 2019). However, in mice fed a HFD, lacking the *Drp1* gene reduced the hepatic fat deposition and ER stress through the expression of *Fgf21*, which plays a beneficial role in mitochondrial dynamics and prevents the release of pro-fibrotic mediators (Wang et al., 2015). It has also been reported that *Drp1* knockdown exacerbated liver fibrosis and inflammation in a mouse model of NASH (Steffen et al., 2022). In hepatocellular carcinoma, Li et al. reported an intense activation of mitochondrial fusion (regulated by the *Opa1* gene) in tumor tissue as well as in organoids from cholangiocarcinoma (Li et al., 2020). The knockdown of *Opa1* inhibited the fusion process in hepatocellular carcinoma cell lines and cholangiocarcinoma tumor organoids (Li et al., 2020). In the present study, the expression of mitochondrial fission protein (DRP1 and MFF) and fusion protein (OPA1) was significantly increased in NLO (Figure 1E). These findings highlight the important role of DRP1 in regulating mitochondrial fission. Thus, the pharmacological inhibition of DRP1 has become a promising therapeutic strategy to ameliorate NASH development.

Mdivi-1, a quinazolinone derivative, has been revealed to play a valuable role in various pathologies via inhibiting DRP1-mediated mitochondrial fission (Manczak et al., 2019). Mdivi-1 attenuated lipopolysaccharide-provoked excessive stimulator of interferon gene activation in Kupffer cells and protected liver function via inhibiting DRP1 (Zhang et al., 2022). In human hepatic organoids, Mdivi-1 mitigated the alcohol-produced mitochondrial retrograde signaling and hepatic steatosis via DRP1 inhibition (Angireddy et al., 2020). In esophageal 3D organoids, the mitochondrial dysfunction-induced cellular transformation was accompanied by elevated DRP1 and its pharmacologic inhibition by Mdivi-1 in MPV17^-/-^ organoids reversed the phenotype to that of normal esophageal epithelial organoids (Guha et al., 2019). In pulmonary arterial hypertension with upregulated DRP1 in fibrotic areas of the right ventricle, treatment with Mdivi-1 improved fibrosis *in vitro* (Tian et al., 2018). In the present study, treatment of NLO with Mdivi-1 decreased significantly the dendritic morphology (Figure 2B) and mRNA expression of *Col1a1* and *Acta2* (Figure 2D) as well as the protein expression of Acta2 in NLO (Figure 2E). Further, Mdivi-1 decreased the oleic acid-induced lipid accumulation in NLO (Supplementary Figures S2A, B).

*In vivo*, long-term administration of Mdivi-1 to NASH model mice suppressed lipid droplet expression in liver tissue (Figures 3 B, C), improved mitochondrial morphological abnormalities (Figure 3C), and induced suppression of ROS production (Figure 5A). It also suppressed the expression of collagen fibers in liver tissue (Figures 4A, B) and protein expression of α-SMA (Figure 4C). Further, it increased the capacity of organoid formation (Figure 5B). These data indicate for the first time that DRP1 is also deeply involved in the development of fibrosis in NASH and that DRP1-mediated mitochondrial fission is important for the regulation of fibrosis pathology. These results suggest that specific inhibition of DRP1 via Mdivi-1 could prevent the development of NASH disease and liver injury. Nevertheless, the lack of specificity of Mdivi-1 towards human *Drp1* may have contributed to paradoxical results of Mdivi-1 in some studies showing no cytoprotective impacts and an increase in cell death (Gharanei et al., 2013; Lin et al., 2015; Ong et al., 2019). Therefore, trials to find more specific *Drp1* inhibitors are still ongoing. Rosdah et al. identified a novel small molecule inhibitor of *Drp1* (Drp1i27) that could directly bind to the human isoform 3 of *Drp1* and increase the cellular networks of fused mitochondria in a dose-dependent way without any effects in *Drp1* knock-out cells (Rosdah et al., 2022). It showed cytoprotective potentials in human fibroblasts exposed to oxidative stress, HL-1 cells with ischemia-reperfusion damage, and human iPSC-derived cardiomyocytes with doxorubicin-induced cytotoxicity (Rosdah et al., 2022). Thus Drp1i27 might be a promising alternative to Mdivi1 for the inhibition of *Drp1*.

The difference in the expression level of *Acta2* between organoids (Figure 2D) and liver tissues (Figure 4C) is attributed to the higher purity of organoid cells than liver tissue cells that contain different kinds of cells (Yin et al., 2016). Thus, we used different analyses to verify the effect of Mdivi-1, where H&E images verify the *in vivo* effect of Mdivi-1 in decreasing collagen fibers (Figure 4A) and lipid droplets (Figure 3B).

Transmembrane 6 superfamily member 2 (*TM6SF2*) was expressed predominantly in the liver and intestines and had an important role in regulating liver fat metabolism via influencing triglyceride secretion and hepatic lipid droplet content (Mahdessian et al., 2014). Several reports obtained from population genetic studies indicated that *TM6SF2* was a risk factor for liver injury and was positively linked with different stages of NASH and fibrosis. (Li et al., 2018; Luo et al., 2022). On the other hand, Newberry et al. demonstrated that liver-specific deletion of *Tm6sf2* promoted steatosis, fibrosis, and hepatocellular cancer in mice by impairing the very low-density lipoprotein secretion (Newberry et al., 2021). *Tm6sf2* knockout in mice enhanced liver triglyceride content threefold and reduced very low-density lipoprotein secretion by 50% (Kozlitina et al., 2014). In the present study, we analyzed genes related to lipid metabolism and found that the expression of *Tm6sf2* was significantly increased in the liver tissues of NASH model mice (Figure 3D). However, we for the first time showed that mice administered Mdivi-1 revealed a significant decrease in expression of *Tm6sf2*. This suggests that Tm6sf2 is involved in fat accumulation in NASH and that targeting *Tm6sf2* is important for improving lipid metabolism and suppressing fat accumulation in NASH.

## 5 Conclusion

**FIGURE 6:**
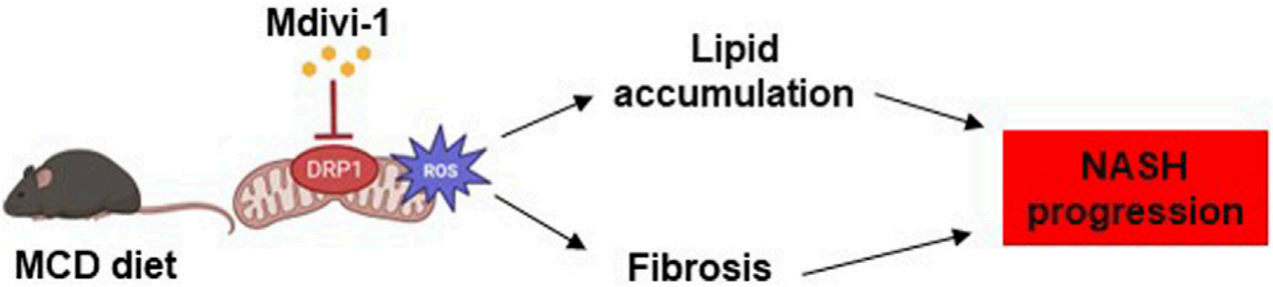
Summary of the effects of Mdivi-1 on NASH progression. In NASH pathology, overexpression of DRP1 in hepatocytes triggers aberrant mitochondrial dynamics, increases ROS production, fat accumulation, and fibrosis. Treatment of NASH organoids or NASH mice with Mdivi-1 reduced ROS production, which might lead to decreased lipid accumulation and fibrosis in the NASH progression.

The results of this study suggest that DRP1, an important therapeutic target in NASH pathology, may regulate abnormal mitochondrial function and morphology, ROS production, and increased oxidative stress leading to fat accumulation and fibrosis (Figure 6). In addition, Mdivi-1 can specifically inhibit the activity of DRP1. Further, the clarification of the detailed mechanisms of action (e.g., loss-of-function studies for *Tm6sf2*) is expected to add more details prior to the application of Mdivi-1 as a therapeutic agent for NASH.

## Data Availability

The original contributions presented in the study are included in the article/Supplementary Materials, further inquiries can be directed to the corresponding authors.
