# Supplementary material for: Evaluation of the efficacy of mitochondrial fission inhibitor (Mdivi-1) using non-alcoholic steatohepatitis (NASH) liver organoids
Source: Front Pharmacol. 2023 Oct 12;14:1243258. doi: 10.3389/fphar.2023.1243258 (PMC10600465; doi:10.3389/fphar.2023.1243258)
Supplement: Supplementary file 1 [file Presentation1.pptx]

## Slide 1
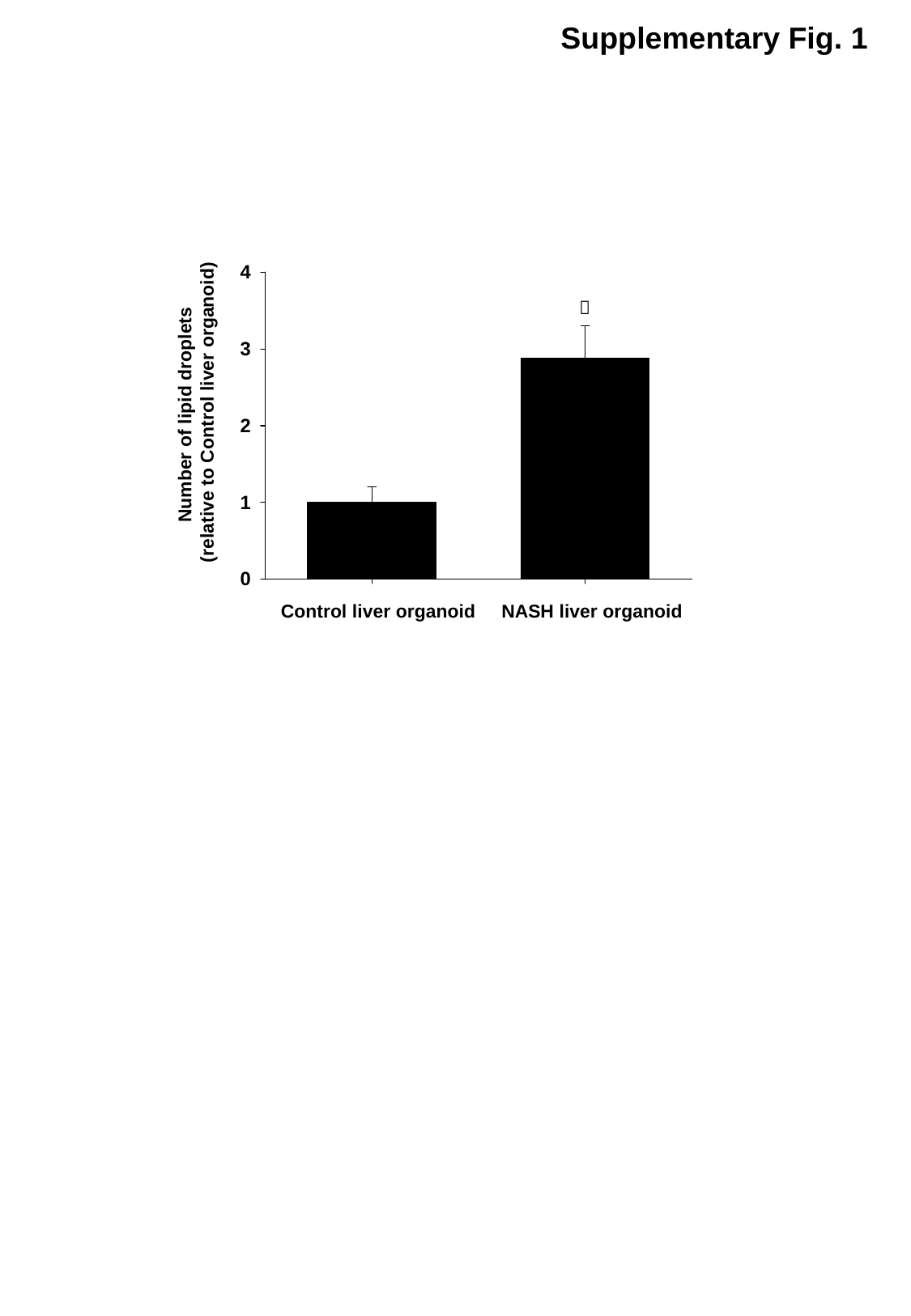

Supplementary Fig. 1
＊
Number of lipid droplets
 (relative to Control liver organoid)
Control liver organoid
NASH liver organoid

## Slide 2
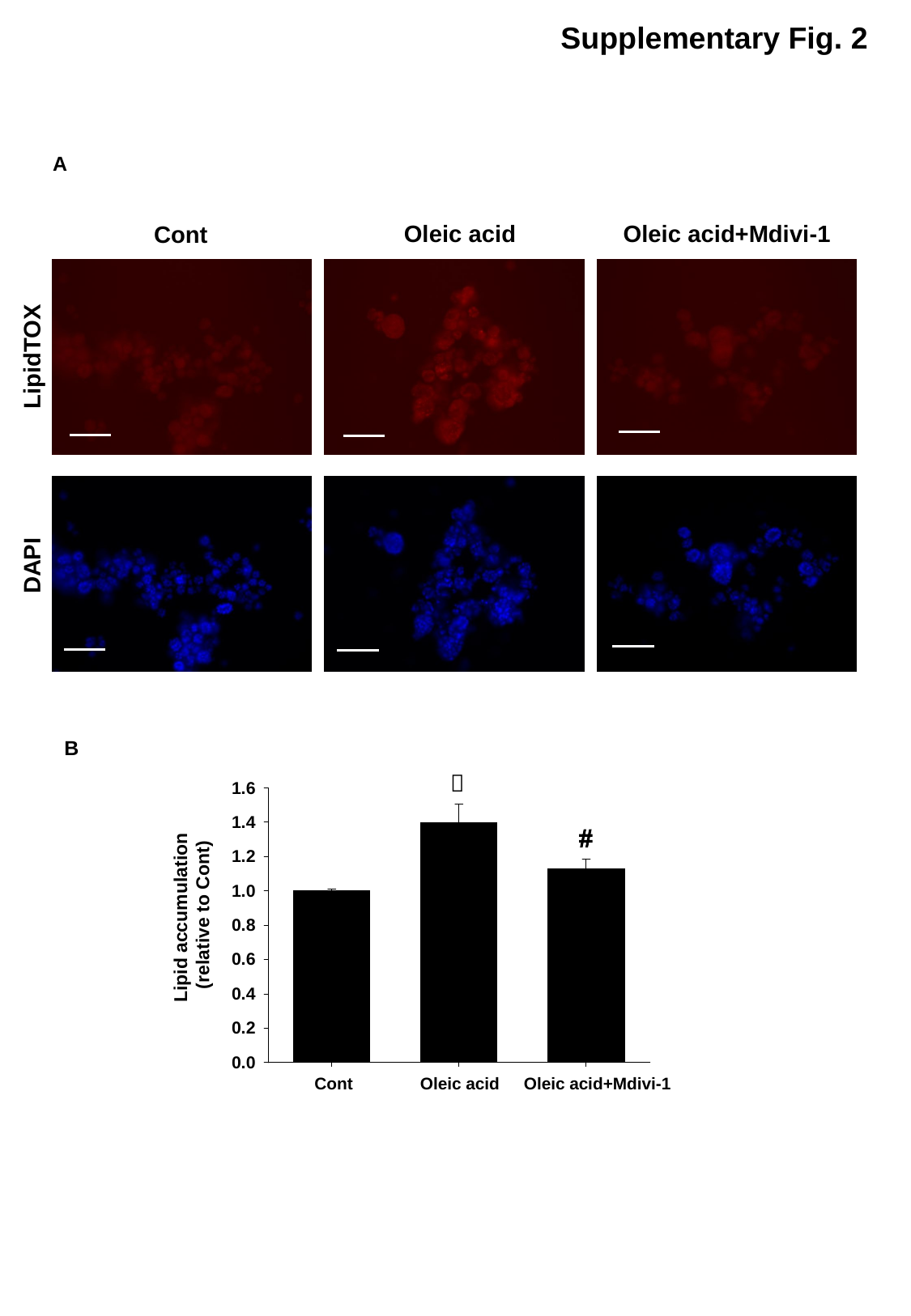

Supplementary Fig. 2
A
Oleic acid+Mdivi-1
Oleic acid
Cont
LipidTOX
DAPI
B
＊
#
Lipid accumulation
 (relative to Cont)
Cont
Oleic acid
Oleic acid+Mdivi-1

## Slide 3
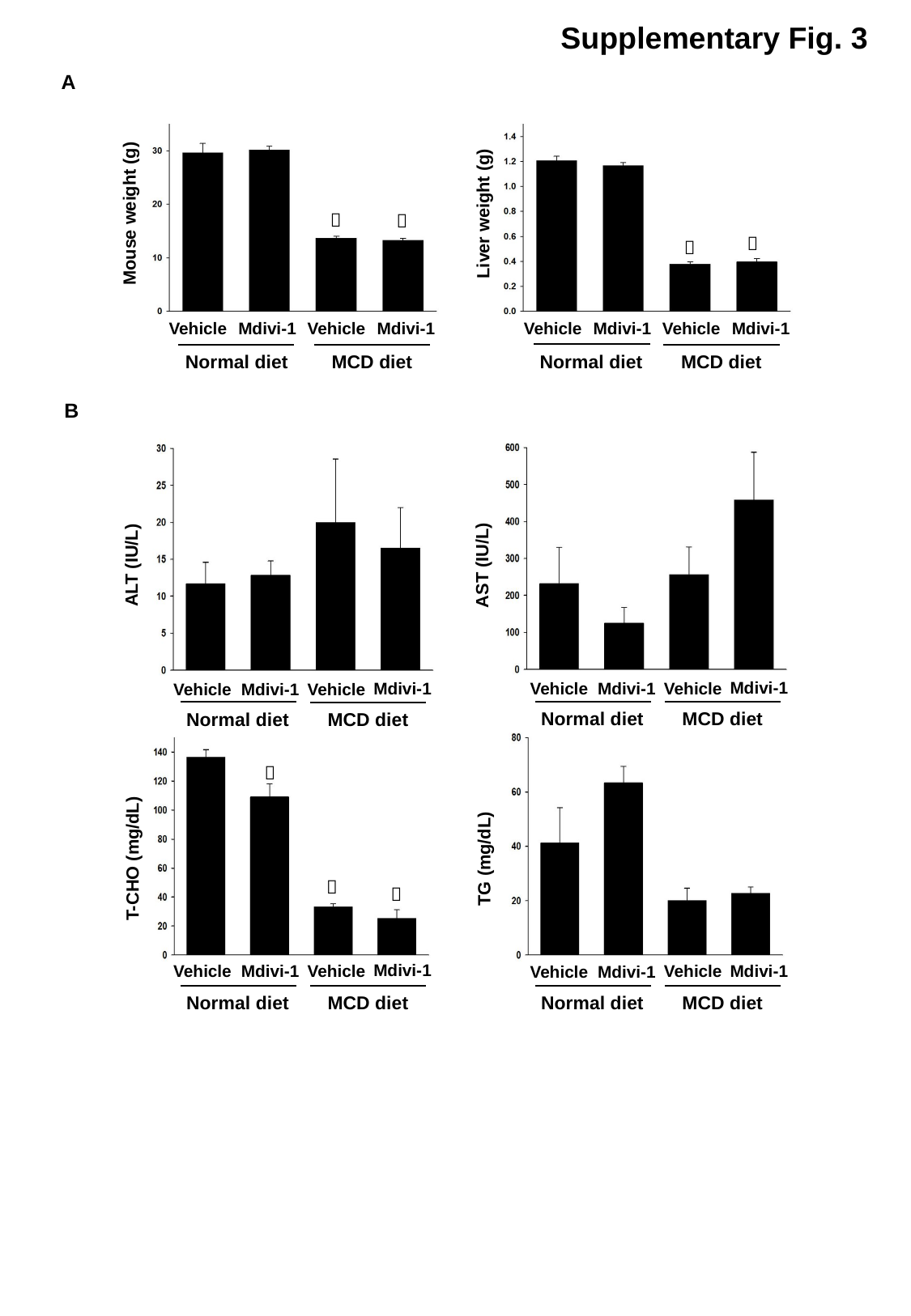

Supplementary Fig. 3
A
Mouse weight (g)
Liver weight (g)
＊
＊
＊
＊
Vehicle
Mdivi-1
Vehicle
Mdivi-1
Vehicle
Mdivi-1
Vehicle
Mdivi-1
Normal diet
MCD diet
Normal diet
MCD diet
B
ALT (IU/L)
AST (IU/L)
Mdivi-1
Vehicle
Mdivi-1
Vehicle
Mdivi-1
Vehicle
Mdivi-1
Vehicle
Normal diet
MCD diet
Normal diet
MCD diet
＊
T-CHO (mg/dL)
TG (mg/dL)
＊
＊
Mdivi-1
Vehicle
Mdivi-1
Vehicle
Mdivi-1
Vehicle
Mdivi-1
Vehicle
Normal diet
MCD diet
Normal diet
MCD diet

## Slide 4
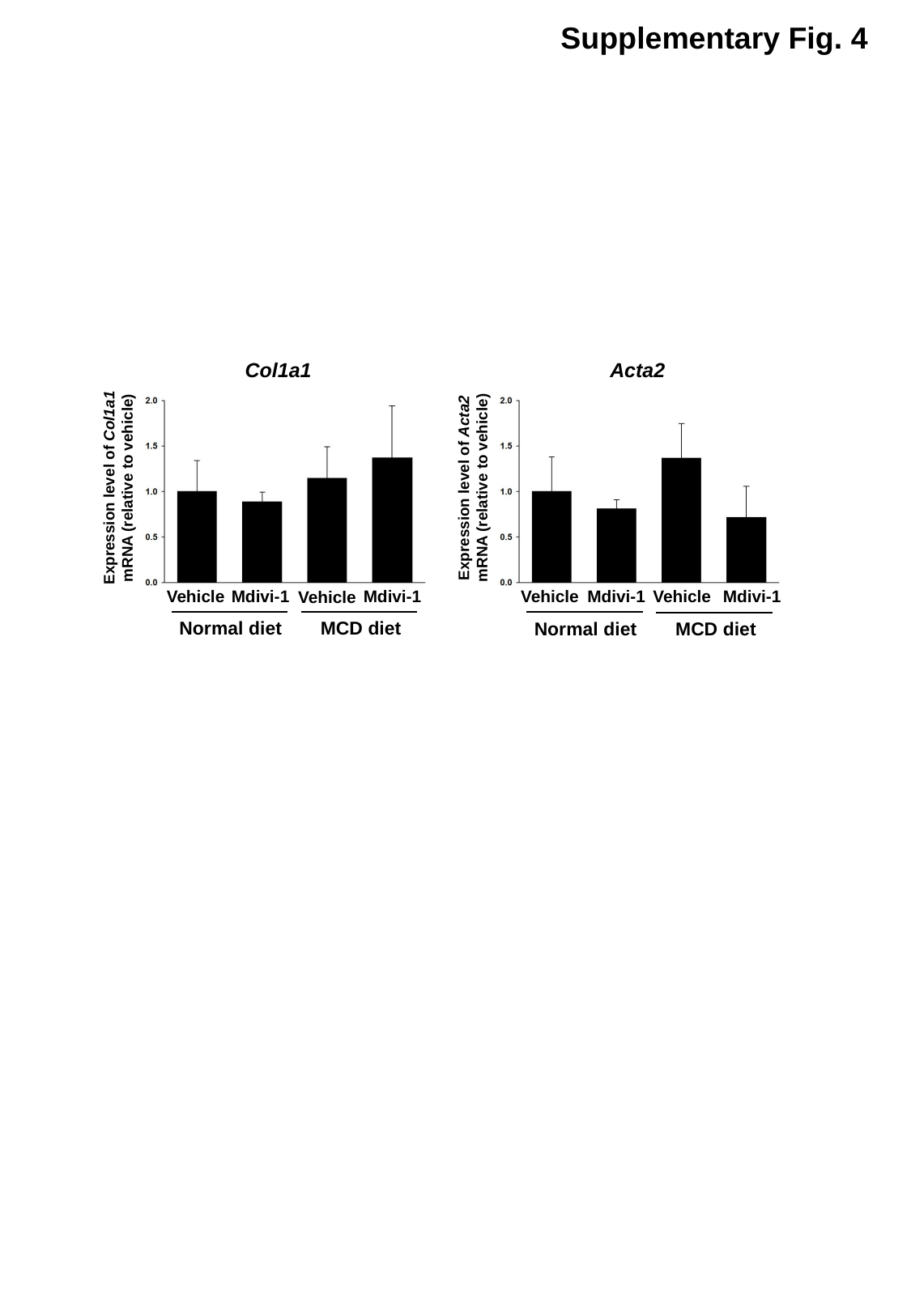

Supplementary Fig. 4
Col1a1
Acta2
Expression level of Col1a1
mRNA (relative to vehicle)
Expression level of Acta2
mRNA (relative to vehicle)
Vehicle
Mdivi-1
Mdivi-1
Vehicle
Vehicle
Mdivi-1
Mdivi-1
Vehicle
Normal diet
MCD diet
Normal diet
MCD diet
