# Supplementary material for: Evaluation of the efficacy of mitochondrial fission inhibitor (Mdivi-1) using non-alcoholic steatohepatitis (NASH) liver organoids
Source: Front Pharmacol. 2023 Oct 12;14:1243258. doi: 10.3389/fphar.2023.1243258 (PMC10600465; doi:10.3389/fphar.2023.1243258)
Supplement: Supplementary file 2 [file DataSheet1.docx]

**Evaluation of the efficacy of the mitochondrial division inhibitor Mdivi-1 using non-alcoholic steatohepatitis (NASH) liver organoids**

^1,2,3^Mohamed Elbadawy*, ^1^Kiwamu Tanabe, ^1^Haru Yamamoto, ^1^Yusuke Ishihara, ^1^Maria Mochizuki, ^1,4^Amira Abugomaa, ^5^Hideyuki Yamawaki, ^6^Masahiro Kaneda, ^1^Tatsuya Usui*, ^1^Kazuaki Sasaki.

^1^Laboratory of Veterinary Pharmacology, Department of Veterinary Medicine, Faculty of Agriculture, Tokyo University of Agriculture and Technology, 3-5-8 Saiwai-cho, Fuchu, Tokyo 183-8509, Japan

^2^Department of Pharmacology, Faculty of Veterinary Medicine, Benha University, 13736, Moshtohor, Toukh, Egypt.

^3^Department of Pathology, College of Veterinary Medicine, University of Georgia, Athens, GA 30602, USA.

^4^Faculty of Veterinary Medicine, Mansoura University, 35516 Mansoura, Egypt

^5^Laboratory of Veterinary Pharmacology, School of Veterinary Medicine, Kitasato University, 35-1 Higashi 23 ban-cho, Towada, Aomori 034-8628, Japan

^6^Laboratory of Veterinary Anatomy, Department of Veterinary Medicine, Faculty of Agriculture, Tokyo University of Agriculture and Technology, 3-5-8 Saiwai-cho, Fuchu, Tokyo 183-8509, Japan

**Corresponding authors:**

**Tatsuya Usui**, DVM, Ph.D., Laboratory of Veterinary Pharmacology, Department of Veterinary Medicine, Faculty of Agriculture, Tokyo University of Agriculture and Technology, 3-5-8 Saiwai-cho, Fuchu, Tokyo 183-8509, Japan. Phone: +81-42-367-5770; FAX: +81-42-367-5770

E-mail: [fu7085@go.tuat.ac.jp](mailto:fu7085@go.tuat.ac.jp)

**Mohamed Elbadawy**, PhD., Department of Pharmacology, Faculty of Veterinary Medicine, Benha University, 13736, Moshtohor, Toukh, Elqaliobiya, Egypt. Laboratory of Veterinary Pharmacology, Department of Veterinary Medicine, Faculty of Agriculture, Tokyo University of Agriculture and Technology, 3-5-8 Saiwai-cho, Fuchu, Tokyo 183-8509, Japan. Phone: +81-42-367-5770

E-mail: [Mohamed.elbadawy@fvtm.bu.edu.eg](mailto:Mohamed.elbadawy@fvtm.bu.edu.eg)

**Supplementary figure legends**

**Supplementary Fig. 1.** Comparison of lipid droplets in non-alcoholic steatohepatitis (NASH) liver organoids (NLO). The number of lipid droplets was counted and shown as a fold increase relative to control liver organoid and expressed as mean ± S.E.M (n=4). **P<*0.05 vs. Control liver organoid.

**Supplementary Fig. 2.** Effects of Mdivi-1 on free fatty acid (FFA)-induced lipid accumulation in NLO. NLO were subjected to 2 mM oleic acid with or without 50 µM Mdivi-1 for 48 h and stained with red LipidTOX and Hoechst dye. Representative images for red LipidTOX staining of vehicle-treated, oleic acid-treated, and oleic acid+Mdivi-1-treated NLO were shown (A, n=4). Scale bar: 100 µm. The fluorescence intensity in the stained images was quantified by using ImageJ software. Results were shown as fold increase relative to the control and expressed as mean ± S.E.M. * *P*<0.05 vs. Cont (vehicle-treated NLO), # *P*<0.05 vs. Oleic acid (oleic acid-treated NLO).

**Supplementary Fig. 3.** Comparison of bodyweight and liver weight in NASH model mice after Mdivi-1 administration. Results were expressed as mean ± S.E.M. **P<*0.05 vs. vehicle with normal diet (A). Comparison of ALT, AST, T-CHO, and TG serum levels as indicators of liver function in NASH model mice after Mdivi-1 administration. Results were expressed as mean ± S.E.M. **P<*0.05 vs. vehicle with normal diet (B).

**Supplementary Fig. 4.** Effects of long-term Mdivi-1 administration to mice on liver fibrosis. Expression of fibrosis-related genes, *Col1a1* and *Acta2* mRNA in liver tissues from each group of mice was determined by quantitative real-time PCR. The expression level of each gene was quantified based on the ratio of expression level to *GAPDH* and shown as a fold increase relative to vehicle with normal diet (n=4-5). Results were expressed as mean ± S.E.M.
